# Pharmacological Strategies to Prevent Contrast-Induced Acute Kidney Injury

**DOI:** 10.1155/2014/236930

**Published:** 2014-02-26

**Authors:** Pattharawin Pattharanitima, Adis Tasanarong

**Affiliations:** Nephrology Unit, Department of Medicine, Faculty of Medicine, Thammasat University (Rangsit Campus), Klong Luang, Pathumtani 12121, Thailand

## Abstract

Contrast-induced acute kidney injury (CI-AKI) is the most common iatrogenic cause of acute kidney injury after intravenous contrast media administration. In general, the incidence of CI-AKI is low in patients with normal renal function. However, the rate is remarkably elevated in patients with preexisting chronic kidney disease, diabetes mellitus, old age, high volume of contrast agent, congestive heart failure, hypotension, anemia, use of nephrotoxic drug, and volume depletion. Consequently, CI-AKI particularly in high risk patients contributes to extended hospitalizations and increases long-term morbidity and mortality. The pathogenesis of CI-AKI involves at least three mechanisms; contrast agents induce renal vasoconstriction, increase of oxygen free radicals through oxidative stress, and direct tubular toxicity. Several strategies to prevent CI-AKI have been evaluated in experimental studies and clinical trials. At present, intravascular volume expansion with either isotonic saline or sodium bicarbonate solutions has provided more consistent positive results and was recommended in the prevention of CI-AKI. However, the proportion of patients with risk still develops CI-AKI. This review critically evaluated the current evidence for pharmacological strategies to prevent CI-AKI in patients with a risk of developing CI-AKI.

## 1. Introduction

Contrast-induced acute kidney injury (CI-AKI) is the most common iatrogenic cause of acute kidney injury (AKI) after intravenous contrast media administration, with an incidence occurring from 1 to 25% [1–4]. CI-AKI has been defined as the acute deterioration of renal function after contrast media administration in the absence of other causes [5]. Unfortunately, the definition of CI-AKI has not been reliable in the literature, which makes the data comparison from various complex studies. In general, CI-AKI was defined as an increase in serum creatinine (SCr) concentration of 0.5 mg/dL or 25% above baseline within 48 h after contrast administration [6–10]. Impairment of renal function in CI-AKI occurs within 3 days after intravenous contrast media administration, while the peak of SCr is observed at 3 to 5 days and returns to the baseline value within 1 to 3 weeks [11, 12]. The incidence of CI-AKI is low (1 to 2%) in patients with normal renal function [1] but increases as high as 25% in high risk patients especially with chronic kidney disease (CKD) or diabetes mellitus with CKD [2, 13]. In addition, old age, higher volume of contrast agent used, congestive heart failure, hypotension, anemia, use of nephrotoxic drug, and volume depletion have been associated with increased risk of CI-AKI [14–16]. Consequently, CI-AKI, particularly in high risk patients, contributes to extended hospitalizations and increases long-term morbidity and mortality [17–19].

Development of CI-AKI involves at least three complementary pathophysiological processes. First, contrast agents induce renal vasoconstriction, accompanied by shunting of blood flow from the medulla to the cortex, a consequence of reducing renal blood flow to the medulla which is followed by renal medulla ischemia [20]. Second, hypoxia can promote further ischemic renal injury by the increase of oxygen free radicals through oxidative stress [21]. Organ injury occurs when tissue hypoperfusion generates reactive oxygen species (ROS) that exceed the patient's antioxidant reserves [22]. Finally, contrast agent is direct tubular toxicity, leading to mitochondrial dysfunction, generation of ROS, and program cell death [6, 22, 23].

In fact, there is no effective therapy once AKI has turned on. Thus, preventive approach should be the best option for all patients with risk to avoid CI-AKI. Several strategies to prevent CI-AKI have been tested in animal models and clinical trials. The rationale for the prevention of CI-AKI by periprocedural intravascular volume expansion is through blocking its two complementary pathophysiological processes [24]. First, expansion of the intravascular space is thought to blunt the vasoconstrictive effect of contrast on the renal medulla. Second, intravascular fluids are believed to attenuate the direct toxic effect of contrast agents on tubular epithelial cells. Hence, intravascular volume expansion with isotonic saline and using of iso-osmolar contrast agents have provided more consistent positive results and were recommended in the prevention of CI-AKI [25, 26]. However, the proportion of patients with risk still develops CI-AKI.

Several pharmacologic agents have been evaluated for the prevention of CI-AKI. The mechanisms of pharmacological prophylaxis for CI-AKI include vasodilator; antioxidant agents have been implicated in the pathogenesis of CI-AKI. This review discusses the current pharmacological strategies to prevent CI-AKI in patients with the risk of developing CI-AKI.

## 2. Pharmacological Strategies to Prevent CI-AKI

### 2.1. Intravascular Volume Expansion for CI-AKI Prevention

The intravascular volume expansion was believed to prevent the adverse effect of contrast media administration by 2 distinct mechanisms: (1) reducing the vasoconstrictive effect of contrast media on renal medulla by suppression of vasopressin secretion, inhibition of renin-angiotensin-aldosterone system, and increase of prostaglandin synthesis, and (2) attenuating the direct toxic effect of contrast media on renal tubular epithelial cells by decreasing proximal tubular salt and water reabsorption which results in diluting the intratubular fluid and reducing the intratubular viscosity [24]. In animal model, the sodium-replete dogs had less magnitude and duration of vasoconstrictive response after contrast media administration than sodium-deplete dogs [27]. A reduction of glomerular filtration rate, renal plasma flow, and alteration of the antioxidant enzyme activity after contrast media administration occurred only in water-depleted rats but not in water-replete rats [28, 29].

The first clinical trial for intravascular volume expansion to prevent CI-AKI was presented in 1994. Solomon et al. [30] randomized 78 patients with CKD who underwent coronary angiography to receive intravenous 0.45% saline alone, for 12 h before and 12 h after the procedure, or in a combination with mannitol or furosemide. The incidence of CI-AKI occurred 11% in 0.45% saline group, 28% in 0.45% saline plus mannitol group, and 40% in 0.45% saline plus furosemide group (*P* = 0.02 for the comparison with the 0.45% saline group). However, the efficacy of intravenous volume expansion to prevent CI-AKI was inconclusive because there was no control or nonsaline infusion group in this study. In addition, the most suitable route of fluid administration and the type of fluid was doubtful.

### 2.2. Oral Fluid versus Intravenous Fluid for CI-AKI Prophylaxis

There are several trials studied on the effects of route of fluid administration on CI-AKI prophylaxis. Taylor et al. [31] randomized 36 patients with CKD who underwent cardiac catheterization to receive intravenous 0.45% saline at a rate of 75 mL/h for 12 h before and 12 h after the procedure or oral hydration at a rate of 1,000 mL over 10 h before the procedure plus intravenous 0.45% saline at a rate of 300 mL/h beginning just before and 6 h after the procedure. The incidences of CI-AKI were not different between intravenous alone and intravenous plus oral fluid group, 6 and 11%, respectively. Trivedi et al. [32] randomized the patients who underwent nonemergency cardiac catheterization to receive intravenous 0.9% saline at a rate of 1 mL/kg/h for 12 h before and 12 h after the procedure or unrestricted oral fluid. The incidences of CI-AKI were significantly higher in intravenous fluid group than in unrestricted oral fluid group, 3.7 and 34.6%, respectively (*P* = 0.005). Dussol et al. [33] randomized 312 patients with CKD who underwent various radiological procedures which required contrast media into 4 groups to receive (1) oral NaCl 1 g per 10 Kg for 2 days before the procedure, (2) intravenous 0.9% saline at a rate of 15 mL/kg/h for 6 h before the procedure, (3) intravenous 0.9% saline at a rate of 15 mL/kg/h for 6 h before the procedure plus theophylline 5 mg/kg 1 h before the procedure, or (4) intravenous 0.9% saline at a rate of 15 mL/kg/h for 6 h before the procedure plus furosemide 3 mg/kg after the procedure. The incidences of CI-AKI were not significantly different between the 4 groups: 6.6, 5.2, 7.5, and 15.2% in groups 1 to 4, respectively. Cho et al. [34] randomized 91 patients with CKD who underwent cardiac catheterization into 4 groups to receive (1) intravenous 0.9% saline 3 mL/kg over 1 h just before and at a rate of 1 mL/kg/h for 6 h after the procedure, (2) intravenous isotonic NaHCO_3_ 3 mL/kg over 1 h just before and at a rate of 1 mL/kg/h for 6 h after the procedure, (3) oral water 500 mL in 2 h which begin 4 h before and 600 mL after the procedure, (4) oral water 500 mL in 2 h which begin 4 h before the procedure with oral NaHCO_3_ 46.4 mEq 20 min before the procedure, and (5) 600 mL of oral water after the procedure with oral NaHCO_3_ 30.4 mEq at 2 and 4 h after the initial dose. The incidences of CI-AKI were not significantly different between the 4 groups: 22, 10, 5, and 5% in groups 1 to 4, respectively. According to these conflicting results, the appropriate route of fluid administration remains inconclusive.

### 2.3. Isotonic Fluid versus Hypotonic Fluid for CI-AKI Prophylaxis

Mueller et al. [25] conducted a study to compare the efficacy of intravenous 0.45% NaCl and 0.9% NaCl for CI-AKI prophylaxis. 1,620 patients who underwent coronary angiography were randomized to receive 0.9% saline or 0.45% saline plus 5% glucose at a rate of 1 mL/kg/h beginning at 8 AM on the day of procedure until 8 AM on the next morning. The incidences of CI-AKI were significantly lower in isotonic saline group than in half-isotonic saline group: 0.7 and 2.0%, respectively (*P* = 0.04). On subgroup analysis, the patients with diabetes received radiocontrast ≥ 250 mL and particularly female patients had benefit from the isotonic fluid therapy.

### 2.4. Sodium Chloride versus Sodium Bicarbonate for CI-AKI Prophylaxis

The administration of intravenous fluid that contains sodium bicarbonate can cause an alkalinization of the intratubular fluid and result in reduction of injurious hydroxyl radicals, which, theoretically, might be more beneficial than sodium chloride fluid therapy in CI-AKI prophylaxis. Merten et al. [35] randomized 119 patients with SCr ≥ 1.1 mg/dL who underwent radiographic procedure requiring contrast media to receive isotonic saline or sodium bicarbonate at a similar rate of 3 mL/kg/h for 1 h before and 1 mL/hg/h for 6 h after the procedure. The incidences of CI-AKI were significantly higher in sodium chloride group than in sodium bicarbonate group: 13.6 and 1.7%, respectively (*P* = 0.02). Briguori et al. randomized 366 patients with CKD who underwent coronary and/or peripheral angiography into 3 groups to receive intravenous (1) 0.9% saline with NAC, (2) sodium bicarbonate with NAC, and (3) 0.9% saline with ascorbic acid and NAC. The incidences of CI-AKI were significantly lower in sodium bicarbonate with NAC group: 9.9, 1.9, and 10.3% in groups 1 to 3, respectively (*P* = 0.019). Recio-Mayoral et al. [36] randomized 111 patients with acute coronary syndrome who underwent emergency percutaneous coronary intervention to receive sodium bicarbonate with NAC or 0.9% saline with NAC. The incidences of CI-AKI were significantly lower in sodium bicarbonate with NAC than in 0.9% saline with NAC group: 1.8 and 21.8%, respectively (*P* < 0.001).

### 2.5. Meta-Analysis Comparing the Efficacy of Sodium Chloride with Sodium Bicarbonate for CI-AKI Prophylaxis

Data from several meta-analyses of the efficacy of sodium chloride versus sodium bicarbonate for CI-AKI prophylaxis are summarized in Table 1. Six out of six meta-analyses [37–42] demonstrated that volume expansion therapy with sodium bicarbonate is superior to sodium chloride in preventing CI-AKI. However, the efficacy of sodium bicarbonate and sodium chloride was not significantly different in meta-analyses from 14 unpublished studies by Zoungas et al. [41] (RR = 0.78, 95% confidence interval 0.52–1.17; *P* = 0.05). The inconsistent data between published and unpublished studies should be cautiously considered in the use of this treatment for CI-AKI prophylaxis until more reliable evidence from large-scale clinical studies is available.

In summary, although the most efficacious route for volume expansion in CI-AKI prophylaxis remains debatable, the intravenous route is more reliable for fluid delivery to the patients. Thus, we suggested using the intravenous route if it is available for CI-AKI prophylaxis. The CI-AKI prevention with isotonic saline is more effective than hypotonic saline. And according to the available data, the volume expansion with saline is at least as effective as sodium bicarbonate for CI-AKI prophylaxis. Thus, we suggested using intravenous/isotonic saline or sodium bicarbonate for volume expansion in CI-AKI prophylaxis depending on the patient's condition.

### 2.6. N-Acetylcysteine (NAC) for CI-AKI Prevention

The possible role of reactive oxygen radicals in the pathogenesis of CI-AKI led to the evaluation of NAC as an antioxidant. The antioxidant effect of NAC relates to both direct free radical scavenging activity and capability to enhance glutathione synthesis [43]. In experimental study, the administration of contrast media results in augmentation of lipid peroxidation marker, reduction of glomerular filtration rate (GFR), and deterioration of tubular structures. In contrast, pretreatment of animals with antioxidants diminishes the hazardous effect of contrast media, including NAC that attenuates the adverse renal effect from contrast media [28, 44–46].

The clinical trials comparing NAC and placebo for prophylaxis of CI-AKI after angiography are shown in Table 2. The first clinical trial for NAC in the prevention of CI-AKI was reported by Tepel et al. [47] in 2000. In this prospective study, 83 patients with CKD who underwent computed tomography (CT) with intravenous contrast media were randomized to receive 600 mg of oral NAC or placebo twice daily for 2 days. All patients received intravenous 0.45% NaCl at a rate of 1 mL/kgBW/h for 12 h before and 12 h after administration of the contrast media. The CI-AKI occurred in 2% of the NAC group compared to 21% in the placebo group (*P* = 0.01). In 2002, Shyu et al. [48] prospectively randomized 121 patients with CKD who underwent a coronary procedure with standard intravascular volume expansion protocol to receive oral NAC or placebo. The CI-AKI occurred in 3.3% in the NAC group, and 24.6% in placebo group (*P* < 0.001). Similarly, the prospective study published by Kay et al. [49] in 2003 demonstrated a significantly lower incidence of CI-AKI in the patients with CKD undergoing elective coronary intervention who receive oral NAC (4%), compared to the placebo group (12%) (*P* = 0.03).

In contrast, several studies failed to demonstrate the benefit of NAC in the prevention of CI-AKI. Webb et al. [50] prospectively randomized 487 CKD patients who underwent cardiac catheterization to receive a single dose of intravenous NAC 500 mg or placebo within 1 h before the administration of contrast media. All patients received 200 mL of intravenous 0.9% NaCl before the procedure, followed by 1.5 mL/kgBW/h for 6 h or until discharge. The incidences of CI-AKI in both groups were similar: 23.3% and 20.7% in NAC and placebo groups, respectively (*P* = 0.57). In 2011, the largest trial of NAC for the prevention of CI-AKI was published by ACT investigators [3]. The 2,308 patients with one or more risk factors for CI-AKI undergoing coronary or peripheral arterial angiography were randomized to receive 4 doses of 600 mg oral NAC or placebo twice daily. All patients received 0.9% NaCl at a rate of 1 mL/kgBW/h from 6 to 12 h before and 6 to 12 h after procedure. The incidences of CI-AKI were similar, 12.7% in both groups (*P* = 1.00).

### 2.7. Systematic Review and Meta-Analysis of the Efficacy of NAC for CI-AKI Prophylaxis

Data from several meta-analyses of the efficacy of NAC for CI-AKI prophylaxis are summarized in Table 3. To date, at least nine out of the 16 meta-analyses have showed beneficial of NAC treatment effect in reducing the incidence of CI-AKI compared to placebo [51–59]. However, disparate results are shown in the remaining studies [3, 60–65]. Various factors may contribute to these inconsistent efficacies of NAC in CI-AKI prophylaxis, including definition of CI-AKI, baseline risk factors, timing and route of NAC administration, dosage of NAC, amount and type of intravenous hydration protocols, volume, type, and route of administration of contrast media, type of performed procedures, and methodological characteristics of trials. In 2008, Kelly et al. [58] conducted a meta-analysis that included 41 studies with a sample size of 3,393 patients. Their results suggested that oral or IV NAC significantly lowered the risk of CI-AKI when compared with intravascular volume expansion with saline alone (relative risk (RR): 0.62, 95% CI: 0.44–0.88). In 2011, ACT investigators [3] encompassed 46 randomized controlled trials comparing NAC with placebo in patients undergoing cardiac or peripheral angiography. The investigators showed that NAC does not reduce the risk of CI-AKI or other clinically relevant outcomes in at risk patients (RR: 1.00, 95% CI: 0.81–1.25; *P* = 0.97).

In summary, the data regarding the efficacy of NAC in CI-AKI prophylaxis remain controversial. However, due to very low toxicity, low cost, and potential benefit of NAC, this medication remains commonly used for the prophylaxis of CI-AKI. We recommend the use of oral NAC at a dose of 600 mg twice daily on the day before and day of the procedure to patients at risk of CI-AKI.

### 2.8. Statins for CI-AKI Prevention

Statins also have the pleiotropic effect, as an anti-inflammatory effect and antioxidant, besides the main inhibitory effect on hydroxymethylglutaryl coenzyme A reductase. In vitro, statins exerted the production of heme oxygenase-1 protein, interfered with NADPH oxidase activity, diminished adhesion molecule expression, and reduced the free radical formation [66–68]. Pretreatment of rats with statin appeared to attenuate the SCr level elevation and lessened the unfavorable histological findings in ischemic reperfusion injury model [68, 69]. Moreover, statin could attenuate CI-AKI in rat model through modulation of oxidative stress and proinflammatory cytokines [70].

The clinical trials comparing efficacy of statins and placebo for prophylaxis of CI-AKI after contrast media administration are shown in Table 4. In 2004, Attallah et al. [71] retrospectively reviewed 1,002 medical records of the patients who started statin in hospital before the cardiac catheterization compared to those who were not administered statin. The baseline characteristics, SCr, GFR, amount of intravenous fluid, and contrast were similar in both groups. The postcatheterization SCr was significantly better in the statin group (*P* < 0.001). The percentages of patients with CI-AKI were 17.2 and 22.3% in the statin and no statin groups, respectively (*P* = 0.028). Khanal et al. [72] published their retrospective study of 29,409 patients undergoing percutaneous coronary intervention who received preprocedure statin and those who did not. The incidences of CI-AKI were 4.37 versus 5.93 (*P* < 0.0001), and those of nephropathy requiring dialysis were 0.32 versus 0.49 (*P* = 0.03) in the patients who received statin and those who did not. In 2009, Xinwei et al. [73] performed the prospective randomized study to test whether the dosage of statins affects the efficacy of CI-AKI prophylaxis. The 284 patients with acute coronary syndrome undergoing coronary angiography were randomized 1 : 1 ratio into simvastatin 20 mg or 80 mg group. All patients were hydrated with intravenous 0.9% NaCl at a rate of 1 mL/kgBW/h for 6 to 12 h before and 12 h after coronary angiography. The incidence of CI-AKI was significantly less in simvastatin 80 mg group compared to 20 mg group: 5.3 versus 15.7%, respectively. This study showed the importance of statins dosage in the efficacy to prevent CI-AKI.

The prospective, randomized, placebo-controlled trial for determining the efficacy of statins in CI-AKI prevention was performed by Jo et al. [74] in 2008. A total of 3,080 patients who underwent coronary catheterization were randomized to receive simvastatin 40 mg or placebo every 12 h for 2 days before the administration of contrast media. All patients were hydrated with intravenous 0.45% NaCl at a rate of 1 mL/kgBW/h for 12 h before and after the procedure. The incidences of CI-AKI in both groups were similar: 2.5 and 3.4% in simvastatin and placebo groups, respectively. Several studies were performed using other statin, atorvastatin, to evaluate effect on CI-AKI prophylaxis. These studies have produced conflicting results [75–78]. Recently, there were 2 randomized controlled trials to determine the efficacy of rosuvastatin in CI-AKI prevention. First, Han et al. [79] randomized 2,998 patients with type 2 DM and CKD who were undergoing coronary or peripheral angiography to receive rosuvastatin 10 mg for 2 days before and 3 days after intervention or standard of care. The incidences of CI-AKI were significantly different: 2.3 and 3.9% in rosuvastatin and control groups, respectively (*P* = 0.01). Leoncini et al. [80] compared the incidence of CI-AKI in 504 patients with non-ST elevated acute coronary syndrome undergoing coronary angiogram who receive rosuvastatin or no statin treatment on the admission. The incidence of CI-AKI was significantly lower in rosuvastatin group than in control group: 6.7 and 15.1%, respectively (*P* = 0.003).

### 2.9. Systematic Review and Meta-Analysis of the Efficacy of Statin for CI-AKI Prophylaxis

Zhang et al. [81] performed a meta-analysis of published randomized clinical trials to determine the efficacy of short-term administration of high-dose statin compared to placebo among patients undergoing catheterization in preventing CI-AKI. From 8 clinical trials including 1,423 patients, the study showed that high-dose statin treatment could decrease the incidence of CI-AKI (RR: 0.51, *P* = 0.001). However, the subgroup analysis showed that the incidence of CI-AKI was not different in the patients with preexisting renal impairment (RR: 0.9, *P* = 0.73). In contrast, the meta-analysis by Zhou et al. [82] including 5 trials with a total of 1,009 patients revealed that short-term, high-dose statin treatment lowered the incidence of AKI in patients with CKD stage 4 and stage 5 in 3 clinical trials, but not in patients with CKD stage 1 to stage 3 in 2 clinical trials. Zhang et al. [83] performed a systematic review and meta-analysis to determine the efficacy of long-term statin pretreatment to prevent the CI-AKI. Among 6 cohort studies, the chronic statin therapy pretreatment had a protective effect against CI-AKI. In contrast, from 6 randomized controlled trials with a total of 1,194 patients, the short-term, high-dose statin pretreatment had a nonsignificant protective effect against CI-AKI (RR 0.7, 95% CI: 0.48–1.02).

In summary, the current data regarding the efficacy of statins in CI-AKI prophylaxis are inconclusive. There is not enough evidence to support the use of statins in radiology patients. In the future, large well-designed studies are needed to address the efficacy of statins and their long-term clinical outcomes.

### 2.10. Ascorbic Acid (Vitamin C) for CI-AKI Prevention

Due to the antioxidant properties of ascorbic acid, the efficacy of ascorbic acid in the prevention of oxidative stress-associated diseases has been extensively studied. In animal model, vitamin C was able to attenuate the pathological process in the postischemic oxidative injuries and gentamicin and cisplatin induced nephrotoxicities [84–86]. In addition, ascorbic acid protected the kidney in CI-AKI rat model against oxidant stress by an antioxidant property [87].

The details of the clinical trials were summarized in Table 5. Spargias et al. [88] prospectively randomized 231 patients who were undergoing coronary angiography to receive oral ascorbic acid 3 g 2 h before and 2 g in the night and in the morning after the procedure or placebo. All patients were hydrated with 0.9% NaCl at a rate of 50–125 mL/h from randomization to at least 6 h after the procedure. The incidences of CI-AKI were 9 and 20% in the ascorbic acid and placebo groups, respectively (*P* = 0.02).

However, the prospective, randomized clinical trials by Boscheri et al. [89], Jo et al. [90], and Zhou and Chen [91] showed the negative results of ascorbic acid in preventing the CI-AKI. Recently, Brueck et al. [92] prospectively randomized 520 patients who were undergoing CAG into 3 groups to receive (1) ascorbic acid 500 mg 24 h and 1 h before procedure, (2) NAC 600 mg 24 h and 1 h before procedure, and (3) placebo. All patients received intravenous 0.9% NaCl at a rate of 1 mL/kgBW/h from 12 h before to 12 h after the procedure. The incidences of CI-AKI were not significantly different. Due to the conflicting results of ascorbic acid in preventing CI-AKI in at risk patients, the use of ascorbic acid for CI-AKI prophylaxis is deniable. 

### 2.11. Tocopherol (Vitamin E) for CI-AKI Prevention

Tocopherol has been widely studied on its antioxidant property [84, 86], while using this agent for CI-AKI prophylaxis might be theoretically possible. A recent experimental study by Kongkham et al. on alpha tocopherol showed the renoprotective effect on the CI-AKI rat model by attenuating renal damage through antioxidant capacity.

The clinical trials on efficacy of using tocopherol for CI-AKI prophylaxis are summarized in Table 6. The first clinical trial in 2009 of Tasanarong et al. [93] randomized 103 patients who were undergoing coronary angiography to receive oral alpha tocopherol 525 IU once daily for 2 days before and on the day of procedure or placebo. All patients received intravenous 0.9% NaCl 1 mL/kgBW/h for 12 h before and 12 h after the angiography. Compared to placebo group, the incidence of CI-AKI was significantly lower in tocopherol group: 5.88 versus 23.08% (*P* = 0.02). In 2013, Tasanarong et al. [94] published a larger trial which enrolled 305 patients to ensure the positivity of the results. The patients who were undergoing elective coronary angiography were prospectively randomized into 3 groups to receive (1) alpha tocopherol 350 mg per day, (2) gamma tocopherol 300 mg per day, or (3) placebo. The prescribed regimen was initiated 5 days before and continued for 2 days after the angiography. All patients received intravenous 0.9% NaCl at a rate of 1 mL/kgBW/h for 12 h before and 12 h after the angiography. The incidences of CI-AKI were lower in both groups of patients who receive tocopherol treatment: 4.9, 5.9, and 14.9%, respectively (*P* = 0.02).

In contrast, a smaller study by Kitzler et al. [95] showed a negative result. Thirty patients who were undergoing computed tomography with nonionic contrast media were randomized to receive oral 1200 mg of NAC, 540 mg of tocopherol emulsion, or placebo. All patients were hydrated with 0.45% NaCl at a rate of 1 mL/kgBW/h for 12 h before and 12 h after the procedure. No patient developed CI-AKI in this study.

Although the positive results of studies make vitamin E become an interesting option for CI-AKI prophylaxis, the sparse studies and inconsistent results cause a reluctance in using it. In the future, large well-designed studies are needed to prove the efficacy of these tocopherols in preventing CI-AKI.

### 2.12. Dopamine for CI-AKI Prevention

The vasoconstrictor effect of contrast media might play an important role in pathogenesis of CI-AKI. The benefit of dopamine might reduce the risk of CI-AKI by causing renal vasodilation and increasing renal blood flow. In animal model, administration of contrast media resulted in suppression of prostacyclin production, diminishing the renal blood flow, augmentation of medullary hypoxic injury, and histological changes at thick ascending limb of Henle's loop [96, 97]. The effect of low-dose dopamine infusion, called renal dose, is believed to cause renal artery vasodilatation. In human, intravenous infusion of dopamine was associated with an increase in renal blood flow in patients with heart failure [98]. These pharmacological properties might be beneficial in the prevention of CI-AKI.

The clinical trials of dopamine use for CI-AKI prophylaxis are summarized in Table 7. Kapoor et al. [99] randomized 40 patients who were undergoing coronary angiography to receive intravenous low-dose dopamine infusion or nothing. The rising in SCr and development of CI-AKI did not occur in any patient who received dopamine infusion. The study by Hans et al. [100] also showed a favorable outcome in the patients who received a dopamine infusion prior to peripheral angiography compared to placebo.

On the other hand, the studies by Abizaid et al. [101] and Stevens et al. [102] failed to demonstrate the benefit of dopamine infusion before the contrast media administration. Moreover, Abizaid et al. [101] showed that the patients who developed CI-AKI and received low-dose dopamine had a higher peak SCr, prolonged course of AKI, and prolonged length of hospital stays than patients who received saline alone. As a result of limited and inconsistent evidence of dopamine for CI-AKI prophylaxis and possibility of adverse outcome in patients who received dopamine treatment, the dopamine treatment for CI-AKI prophylaxis remains undesirable.

### 2.13. Fenoldopam for CI-AKI Prevention

Fenoldopam is a selective dopamine A1 receptor agonist and hypothetically increases renal blood flow in a similar manner to dopamine. This effect might be beneficial in the prevention of CI-AKI.

The clinical trials of fenoldopam use for CI-AKI prophylaxis are summarized in Table 8. In the first clinical trial [103], the patients were randomized to receive 0.45% NaCl alone or with fenoldopam or NAC. The incidences of CI-AKI were similar: 15.3, 15.7, and 17.7%, respectively (*P* = 0.919). Stone et al. [104] conducted a larger prospective trial comparing patients who received fenoldopam in conjunction with 0.45% NaCl or 0.45% NaCl alone. There was no difference in CI-AKI incidence: 33.6 versus 30.1%, respectively (*P* = 0.61). Ng et al. [105] compared the patients who received intravascular volume expansion protocol with fenoldopam or NAC. There was no difference in the incidence of CI-AKI: 20% versus 11.4%, respectively (*P* = 0.4). Moreover, the administration of fenoldopam resulted in decrease in blood pressure and increase in heart rate [104] which might be potentially harmful to the patients. In summary, all available evidence showed the negative results and undesirable side effect. Hence, the prophylactic use of fenoldopam for CI-AKI is disagreeable. 

### 2.14. Theophylline for CI-AKI Prevention

In general, adenosine is an intrarenal vasoconstrictor and a mediator of the tubuloglomerular feedback mechanism. Theophylline, an adenosine antagonist, was logical to evaluate for risk reduction in CI-AKI. In animal model, the administration of contrast media resulted in an increased excretion of endogenous adenosine. Theophylline is an adenosine antagonist which might theoretically improve the renal hemodynamic in patients who receive contrast media. In experimental study, the decline of renal blood flow after contrast media administration was attenuated by theophylline [106].

The clinical trials of theophylline use for CI-AKI prophylaxis are summarized in Table 9. Two randomized studies by Huber et al. [107, 108] in 2002 and 2003 for evaluation the efficacy of theophylline versus placebo gave positive results. The incidence of CI-AKI was lower in the patients who receive theophylline. The more recent randomized studies also compared the efficacy of theophylline with saline, NAC with saline, and saline alone [109–112]. The incidence of CI-AKI was lower in theophylline group compared to saline group. Moreover, these studies showed comparable [109, 111] or even more preferable [110, 112] results of theophylline than NAC. However, Abizaid et al. [101] randomized 60 patients into 3 groups: (1) aminophylline with saline, (2) saline alone, and (3) dopamine with saline. The incidences of CI-AKI were similar: 35, 30, and 30%, respectively (*P* = 0.6). However, the requirement of RRT was slightly higher among the patients who received aminophylline with saline compared to others: 5 versus 0%.

Ix et al. [113] performed a meta-analysis including 7 trials with 480 patients and showed that mean change of SCr was lower in theophylline and aminophylline pretreatment group (*P* = 0.004). In 2012, Dai et al. [114] conducted a meta-analysis to determine the efficacy of theophylline in CI-AKI prevention. Sixteen trials with 1,412 patients were included. The study showed that theophylline significantly reduced the risk of CI-AKI (RR: 0.48, 95% CI: 0.26–0.89; *P* = 0.02). In contrast, Bagshaw and Ghali [115] published systematic review and meta-analysis and showed that pretreatment with theophylline had a trend toward reduction in CI-AKI incidence (RR: 0.40, 95% CI: 0.14–1.16; *P* = 0.09). Meta-analysis by Kelly et al. [58] with 531 patients from 6 trials showed a nonsignificant protective trend of theophylline for CI-AKI prevention (RR: 0.49, 95% CI: 0.23–1.06).

Due to inconsistent efficacy of theophylline across studies, the use of theophylline for CI-AKI prevention is not suggested.

### 2.15. Nebivolol for CI-AKI Prevention

Nebivolol is a *β*_1_ receptor antagonist which has vasodilatory and antioxidant effect [116, 117]. After contrast media administration, the pretreated rats with nebivolol had less oxidative stress marker and histological abnormalities compared to those without nebivolol pretreatment [118]. 

Table 10 shows the details of clinical trials of nebivolol for CI-AKI prophylaxis. In 2011, Avci et al. [119] prospectively randomized 90 patients undergoing coronary angiogram to receive nebivolol 5 mg once daily with saline or metoprolol 50 mg once daily with saline. The incidence of CI-AKI was significantly lower in nebivolol group: 24 versus 33% (*P* = 0.039), respectively. Günebakmaz et al. [120] randomized 120 patients who were undergoing coronary angiography into 3 groups: (1) nebivolol 5 mg once daily with saline, (2) saline alone, and (3) NAC with saline. The incidence of CI-AKI was numerically lower in nebivolol group: 20, 27.5, and 22.5% (*P* = 0.72), respectively. In behalf of scanty studies in human, the use of nebivolol for CI-AKI prophylaxis is discouraged.

### 2.16. Atrial Natriuretic Peptide for CI-AKI Prevention

Atrial natriuretic peptide (ANP) is a potent endogenous natriuretic compound produced by cardiac myocytes in right atrium. In rat model, ANP infusion results in augmentation of glomerular filtration rate predominantly by a hemodynamic mechanism [121]. ANP treatment showed to ameliorate ischemic AKI in rat [122] and prevent CI-AKI in heart failure induced dogs [123].

Kurnik et al. [124] randomized 247 patients with CKD who were undergoing radiographic procedures which required contrast media administration to receive intravenous 0.45% saline for 12 h before and after the procedure or a combination of saline and one among three different rates of ANP infusion (0.01 *μ*g/kg/min, 0.05 *μ*g/kg/min, or 0.1 *μ*g/kg/min) for 30 min before and continuing for 30 min after the procedure. The incidences of CI-AKI were not different between 4 groups of patients. Morikawa et al. [125] randomized 254 patients with CKD who were undergoing coronary angiography to receive either ANP intravenous infusion at a rate of 0.042 *μ*g/kg/min or intravenous ringer solution alone at a rate of 1.3 mL/kg/h before and after the administration of contrast media. The incidences of CI-AKI were significantly lower in the ANP treatment group than in control group: 3.2 and 11.7%, respectively (*P* = 0.015). At 1 month, the incidences of an increase in SCr of ≥25% or ≥0.5 mg/dL from baseline were also significantly lower in ANP-treated group than in the control group: 2.4 and 12.5%, respectively (*P* = 0.006). Due to sparse in number of evidences and inconsistent efficacies of ANP across the studies, the use of ANP for CI-AKI prevention is not indicative.

### 2.17. Prostaglandins for CI-AKI Prevention

Prostaglandins (PG) arise from enzymatic metabolism of arachidonic acid, which appeared in various parts of the kidney and had an effect on controlling renal blood flow and glomerular filtration rate [126]. In animal model, the vasodilatory effect of PG had an important role in maintaining blood flow to the poorly oxygenated region of the kidney [127], which directly counteracts the renal vasoconstrictive effect after the contrast media administration. Besides, an inhibition of PG synthesis in rats appeared to aggravate the renal injury from contrast media administration [16]. The infusion of PG had protective effects on renal function in either ischemia-reperfusion injury or contrast media administration model [128–130].

Gurkowski et al. [131] randomized 125 patients who were undergoing a radiologic contrast procedure to receive misoprostol, a synthetic PGE_1_ analogue, 200 mg 4 times a day for 3 days before and 2 days after the procedure or a placebo. Misoprostol treatment showed to significantly attenuate the reduction of creatinine clearance. Spargias et al. [132] randomized 208 patients with CKD who were undergoing coronary angiography to receive iloprost, a synthetic analogue of PGI_2_, 1 ng/kg/min for 30–90 minutes before and 4 h after the procedure or placebo. The incidences of CI-AKI were lower in iloprost group than in control group: 8 and 22%, respectively (*P* = 0.005). Despite the positive results of the studies using PG analogue for CI-AKI prophylaxis, the sparse number of studies causes a reluctance in using it. Further studies are needed to prove the efficacy of PG analogue for CI-AKI prophylaxis.

## 3. Conclusion

CI-AKI is a common condition that is associated with increased morbidity and mortality, particularly in high risk patients. Volume expansion and treatment of dehydration are established interventions in the prevention of CI-AKI. Oral volume expansion has demonstrated some benefit, but there is not enough evidence to show that it is as effective as intravenous volume expansion. However, only intravascular volume expansion with isotonic saline solution or sodium bicarbonate is regarded as the only effective therapy and is recommended in the prevention of CI-AKI depending on the patient's volume status assessment. For isotonic saline administration, most studies suggest that 0.9% saline should be started at a rate ≥ 1–1.5 mL/kg/h 3–12 h before and 6–12 h after contrast media exposure. Instead of sodium bicarbonate administration, most studies suggest that sodium bicarbonate should be started at a rate of 3 mL/kg/h 1 h before and 1 mL/kg/h 6 h after contrast media exposure.

There are varieties of pharmacological interventions for CI-AKI prophylaxis that has been developed in many experimental studies and clinical trials. Based on the evidence tables and even taking the most recent study, there are no currently approved pharmacologic agents for the prevention of CI-AKI. Overall evidence of NAC is not consistent or overwhelming, but oral NAC has a low risk of adverse events and usually a low cost. We suggest using oral NAC combined with standard intravenous volume expansion in patients with increased risk of CI-AKI. Recent clinical trials for early high-dose or short-term statin demonstrated the benefit for preventing CI-AKI. In the future, large, well-designed, and adequately powered randomized clinical trials are urgently needed to study this important issue. Other agents, theophylline, nebivolol, prostaglandin, ANP, dopamine, and fenoldopam, showed some benefit reports, but the majority of evidence showed conflicting results and some therapies were even harmful. In addition, the novel pharmacological strategies such as ascorbic acid and tocopherol are required to prove their benefit in preventing CI-AKI in the future. 

Future approaches include large plan excellent clinical trials of oral or intravenous antioxidants, vasodilators, or novel pharmacologic agents combined with intravenous volume expansion to decrease the incidence of CI-AKI. Newer criteria for early diagnosis of CI-AKI by rising SCr, changing urine output, and/or novel biomarker need to be developed and used to be the standard criteria for general practices.

## Figures and Tables

**Table 1:** Meta-analysis comparing the efficacy of sodium bicarbonate and sodium chloride for contrast-induced AKI prophylaxis.

References		Number of patients	Number of trials	RR	95% CI		*P* value
Authors	Year				Low	High	
Hogan et al. [38]	2008	1,307	7	0.37	0.18	0.714	0.005
Kanbay et al. [39]	2009	2,448	17	0.54	0.36	0.83	ND
Navaneethan et al. [40]	2009	1,652	12	0.46	0.26	0.82	0.008
Zoungas et al. [41]	2009						
(i) Published studies		1,846	9	0.43	0.25	0.75	0.02
(ii) Unpublished studies		1,717	14	0.78	0.52	1.17	0.05
Kunadian et al. [42]	2011	1,734	7	0.33	0.16	0.69	0.003
Jang et al. [37]	2012	3,609	19	0.56	0.36	0.86	0.008

**Table 2:** Prospective, randomized clinical trials comparing N-acetylcysteine with placebo for prophylaxis of contrast-induced AKI after angiography.

Authors	Year	Inclusion criteria	Type of procedure and contrast media	Number of patients Intervention versus control	Study protocol		Intravascular volume expansion protocol	CI-AKI definition	Incidence of CI-AKI		RRT requirement Intervention versus control (%)
					Intervention	Control			Intervention versus control (%)	*P* value	
Tepel et al. [47]	2000	Cr >1.2 mg/dL or GFR <50 mL/min/1.73 m^2^	CECT Iopromide	41 versus 42	NAC 600 mg po bid	None	N/2 1 mL/kg/h 12 hours before and after	↑Cr ≥25%/2 d or ↑Cr ≥0.5/2 d	2 versus 21	0.01	0 versus 0

Shyu et al. [48]	2002	Cr 2–6 mg/dL or GFR 8–40 mL/min/1.73 m^2^	CAG Iopamidol	60 versus 61	NAC 400 mg po bid	Placebo	0.45% NaCl 1 mL/kg/h 12 hours before and after	↑Cr ≥0.5/2 d	3.3 versus 24.6	<0.001	ND

Kay et al. [49]	2003	Cr >1.2 mg/dL or GFR <60 mL/min/1.73 m^2^	CAG Iopamidol	102 versus 98	NAC 600 mg po bid	Placebo	NSS 1 mL/kg/h 12 hours before and 6 hours after with liberal oral fluid	↑Cr ≥25%/2 d	4 versus 12	0.03	ND

Baskurt et al. [110]	2009	GFR 30–60 mL/min/1.73 m^2^	CAG Ioversol	73 versus 72 versus 72	NAC 600 mg po bid	(1) None (2) NAC 600 mg and theophylline 200 mg po bid	NSS 1 mL/kg/h 12 hours before and after	↑Cr ≥0.5/2 d	9.6 versus 6.9 versus 0	0.033	0 versus 0

Boccalandro et al. [133]	2003	Cr >1.2 mg/dL or GFR <50 mL/min/1.73 m^2^	CAG Iodixanol	75 versus 106	NAC 600 mg po bid	None	0.45% NaCl 75 mL/h 12 hours before and after	↑Cr ≥0.5/2 d	13 versus 12	0.842	ND

Webb et al. [50]	2004	GFR <50 mL/min/1.73 m^2^	CAG Ioversol	242 versus 245	Single dose of NAC 500 mg in D5W 50 mL IV 1 h before	D5W 50 mL	NSS 200 mL before and 1.5 mL/kg 6 hours after	↓GFR ≥5 mL/min/1.73 m^2^	23.3 versus 20.7	0.51	20 versus 0

Gomes et al. [134]	2005	Cr >1.2 mg/dL	CAG Ioxaglate	77 versus 79	NAC 600 mg po bid 2 doses before and after	Placebo	NSS 1 mL/kg/h 12 hours before and after	↑Cr ≥0.5/2 d	10.4 versus 10.1	1.00	2.6 versus 0

Ozcan et al. [135]	2007	Cr 1.2–4 mg/dL	CAG Ioxaglate	88 versus 88 versus 88	NAC 600 mg po bid with NSS 1 mL/kg/h 6 hours before and after	1 mL/kg/h for 6 hours before and after of (1) Isotonic NaHCO_3_ IV (2) NSS IV	—	↑Cr ≥25%/2 d or ↑Cr ≥0.5/2 d	12.5 versus 4.5 versus 13.6	0.706 0.081	0 versus 1.1 versus 1.1

ACT investigators [3]	2011	At least 1 risk factor for CI-AKI (Age >70 years, Cr >1.5 mg/dL, DM, CHF, LVEF <0.45, hypotension)	CAG/PAG ND	1,172 versus 1,136	NAC 600 mg po bid	Placebo	NSS 1 mL/kg/h × 6–12 hours before and after	↑Cr 25%/2–4 d	12.7 versus 12.7	0.97	2.2 versus 2.3

bid: twice daily; CAG: coronary angiography; CECT: contrast enhanced computed tomography; CI-AKI: contrast-induced acute kidney injury; CHF: congestive heart failure; Cr: creatinine; d: day; DM: diabetes mellitus; D5W: 5% dextrose solution; GFR: glomerular filtration rate; h: hour; IV: intravenous; kg: kilogram body weight; LVEF: left ventricular ejection fraction; mg: milligram; mL: milliliter; NAC: N-acetylcysteine; ND: no data; NSS: normal saline solution; N/2: 0.45% NaCl; PAG: peripheral angiography; po: per oral route; RRT: renal replacement therapy.

**Table 3:** Systematic review and meta-analysis comparing the efficacy of N-acetylcysteine and placebo for contrast-induced AKI prophylaxis.

References		Number of patients	Number of trials	RR	95% CI		*P* value
Authors	Year				Low	High	
Birck et al. [51]	2003	805	7	0.435	0.215	0.879	0.02
Isenbarger et al. [52]	2003	805	7	0.37	0.16	0.84	ND
Alonso et al. [53]	2004	885	8	0.41	0.22	0.79	0.007
Bagshaw and Ghali [54]	2004	1,261	14	0.54	0.32	0.91	0.02
Pannu et al. [55]	2004	1,776	15	0.65	0.43	1.0	0.049
Duong et al. [56]	2005	1,584	14	0.57	0.37	0.84	0.01
Liu et al. [57]	2005	1,028	9	0.43	0.24	0.75	ND
Kelly et al. [58]	2008	6,379	41	0.62	0.44	0.88	ND
Kwok et al. [59]	2013	15,976	7	0.65	0.48	0.88	ND
Kshirsagar et al. [60]	2004	1,538	16	ND	ND	ND	ND
Mirsa et al. [61]	2004	ND	27	ND	ND	ND	NS
Nallamothu et al. [62]	2004	2,195	21	0.73	0.52	1.0	0.08
Zagler et al. [63]	2006	1,892	13	0.68	0.46	1.02	0.06
Gonzales et al. [64]	2007	2,476	22	0.87	0.68	1.12	0.28
ACT Investigators [3]	2011	1,000	5	1.05	0.73	1.53	ND
Sun et al. [65]	2013	1,916	10	0.68	0.46	1.02	0.06

95% CI: 95% confidence interval; ND: no available data; NS: nonsignificant; RR: relative risk.

**Table 4:** Prospective, randomized clinical trials comparing efficacy of statins and placebo for contrast-induced AKI after angiography prophylaxis.

Authors	Year	Type of procedure and contrast media	Number of patients Intervention versus control	Study protocol		Intravascular volume expansion and NAC protocol	CI-AKI definition	Mean GFR Intervention versus control (mL/min/1.73 m^2^)	Incidence of CI-AKI		RRT requirement Intervention versus control (%)
				Intervention	Control				Intervention versus control (%)	*P* value	
Xinwei et al. [73]	2009	CAG Iodixanol	115 versus 113	Simvastatin 20 mg/d	Simvastatin 80 mg/d before and 20 mg after	NSS 1 mL/kg/h 6–12 hours before and 12 hours after	↑Cr ≥25%/2 days or ↑Cr ≥0.5/2 days	86.5 versus 93.6	15.7 versus 5.3	<0.05	ND

Patti et al. [77]	2011	CAG Iobitridol	120 versus 121	Atorvastatin 80 mg 12 hours and 40 mg 2 hours before	Placebo	NSS 1 mL/kg/h ≥ 12 hours before and 24 hours after	↑Cr ≥25%/1-2 days or ↑Cr ≥0.5/1-2 days	79.8 versus 77.0	5 versus 13.2	0.046	0 versus 0.8

Quintavalle et al. [78]	2012	CAG Iodixanol	202 versus 208	Atorvastatin 80 mg 1 days before	None	Isotonic NaHCO_3_ 3 mL/kg/h 1 hour and 1 mL/kg/h 6 hours NAC 1200 mg po bid	↑CysC ≥10%/1 day ↑Cr ≥25%/2 days or ↑Cr ≥0.5/2 days	42 versus 43	4.5 versus 17.8	0.005	ND

Jo et al. [74]	2008	CAG Iodixanol	124 versus 123	Simvastatin 40 mg po q12h for 2 days	Placebo	N/2 1 mL/kg/h 12 hours before and after	↑Cr ≥25%/2 days or ↑Cr ≥0.5/2 days	53.46 versus 55.4	2.5 versus 3.4	1.0	0 versus 0.8

Özhan et al. [75]	2010	CAG ND	Total 130	Atorvastatin	None	NAC	ND	ND	2 patients versus 7 patients*	NS	ND

Toso et al. [76]	2010	CAG Iodixanol	152 versus 152	Atorvastatin 80 mg/d 2 days before and after	Placebo	NSS 1 mL/kg/h 12 hours before and after NAC 1200 mg po bid	↑Cr ≥0.5/5 days or ↑Cr ≥25%/5 days	46 versus 46	9.7 versus 11.2	NS NS	0 versus 0.7

Han et al. [79]	2013	CAG/PAG Iodixanol	1498 versus 1500	Rosuvastatin 10 mg/d 2 days before and 3 days after	None	NSS 1 mL/kg/h 12 hours before and 24 hours after	↑Cr ≥25%/3 days or ↑Cr ≥0.5/3 days	74.16 versus 74.43	2.3 versus 3.9	0.01	0 versus 0.1

Leoncini et al. [80]	2013	CAG Iodixanol	252 versus 252	Rosuvastatin 40 mg on admission then 20 mg/d	None	NSS 1 mL/kg/h 12 hours before and after NAC 1200 mg po bid	↑Cr ≥25%/3 days or ↑Cr ≥0.5/3 days	82.5 versus 82.6	6.7 versus 15.1	0.003	0 versus 0.1

*The incidences of CI-AKI data in each group are not available. Data is shown as the number of patients who develop CI-AKI.

bid: twice daily; CAG: coronary angiography; CI-AKI: contrast-induced acute kidney injury; Cr: creatinine; CysC: cystatin C; d: day; h: hour; IV: intravenous; kg: kilogram body weight; mg: milligram; mL: milliliter; NAC: N-acetylcysteine; ND: no data; NSS: normal saline solution; N/2: 0.45% NaCl; po: per oral route; PAG: peripheral angiography; q12h: every 12 hours; RRT: renal replacement therapy.

**Table 5:** Prospective, randomized clinical trials comparing efficacy of vitamin C with placebo and other agents for contrast-induced AKI after angiography prophylaxis.

Authors	Year	Type of procedure and contrast media	Number of patients Intervention versus control	Study protocol		Intravascular volume expansion and NAC protocol	CI-AKI definition	Mean GFR Intervention versus control (mL/min/1.73 m^2^)	Incidence of CI-AKI		RRT requirement Intervention versus control (%)
				Intervention	Control				Intervention versus control (%)	*P* value	
Spargias et al. [88]	2004	CAG LONICM or IONICM	118 versus 113	Ascorbic acid 3 g po 2 hours before and 2 g in the night and in the morning after	Placebo	NSS 50–125 mL/h from randomization to 6 hours after	↑Cr ≥25%/2 days or ↑Cr ≥0.5/2 days	61.1 versus 68.1	9 versus 20	0.02	ND

Boscheri et al. [89]	2007	CAG ND	74 versus 69	Ascorbic acid 1 g	Placebo	NSS before and after	ND	ND	6.8 versus 4.3	NS	ND

Jo et al. [90]	2009	CAG Iodixanol	106 versus 106	Ascorbic acid po q12h 3 and 2 g before and 2 and 2 g after	NAC 1200 mg po bid 4 doses, begin 1st dose in the evening before	N/2 1 mL/kg/h 12 hours before and after	↑Cr ≥25%/2 days or ↑Cr ≥0.5/2 days	53.7 versus 53.7	4.4 versus 1.2	0.370	2 versus 1

Zhou and chen [91]	2012	CAG Unspecified	74 versus 82	Ascorbic acid 3 g IV before and 0.5 g po q12h for 2 days after	Placebo	NSS 1 mL/kg/h 4 hours before and 12 hours after	↑Cr ≥25%/2 days or ↑Cr ≥0.5/2 days	52.5 versus 53.2	6.3 versus 5.4	0.69	ND

Brueck et al. [92]	2013	CAG Iopromide	104 versus 208 versus 208	Ascorbic acid 500 mg IV at 24 hours and 1 hour before	(1) NAC 600 mg iv at 24 hours and 1 hour before (2) Placebo	NSS 1 mL/kg/h 12 hours before and 12 hours after	↑Cr ≥0.5/3 days	43.0 versus 40.2 versus 42.0	24.5 versus 27.6 versus 32.1	0.11* 0.20**	0 versus 0 versus 0

*P* value of *ascorbic acid and **NAC compared to placebo group.

bid: twice daily; CAG: coronary angiography; CI-AKI: contrast-induced acute kidney injury; Cr: creatinine; g: gram; h: hour; IONICM: isoosmolarity nonionic contrast media; IV: intravenous; kg: kilogram body weight; LONICM: low osmolarity nonionic contrast media; kg: kilogram body weight; mg: milligram; mL: milliliter; NAC: N-acetylcysteine; ND: no data; NSS: normal saline solution; N/2: 0.45% NaCl; po: per oral route; q12h: every 12 hours; RRT: renal replacement therapy.

**Table 6:** Prospective, randomized clinical trials comparing efficacy of vitamin E with placebo and other agents for contrast-induced AKI after angiography prophylaxis.

Authors	Year	Type of procedure and contrast media	Number of patients Intervention versus control	Study protocol		Intravascular volume expansion and NAC protocol	CI-AKI definition	Mean GFR Intervention versus control (mL/min/1.73 m^2^)	Incidence of CI-AKI		RRT requirement Intervention versus control (%)
				Intervention	Control				Intervention versus control (%)	*P* value	
Tasanarong et al. [93]	2009	CAG Iopromide	51 versus 52	Alpha tocopherol 525 IU po OD for 2 days before	Placebo	NSS 1 mL/kg/h 12 hours before and after	↑Cr ≥25%/2 days or ↑Cr ≥0.5/2 days	41 versus 42	5.88 versus 23.08	0.02	0 versus 0

Tasanarong et al. [94]	2013	CAG Iopromide	102 versus 102 versus 101	Po 5 days before and 2 days after of (1) *α*-tocopherol 350 mg/d (2) *γ*-tocopherol 300 mg/d	Placebo	NSS 1 mL/kg/h 12 hours before and after	↑Cr ≥25%/2 days or ↑Cr ≥0.5/2 days	45 versus 46 versus 43	4.9 versus 5.9 versus 14.9	0.02	0 versus 0 versus 0

Kitzler et al. [95]	2012	CT Iopromide	10 versus 10 versus 10	Vitamin E emulsion 540 mg IV every 6 hours 12 hours before and after	(1) NAC 1200 mg po every 6 hours 12 hours before and after plus placebo (2) Placebo	N/2 1 mL/kg/h 12 hours before and after	↑Cr ≥25%/2 days	64 versus 56 versus 63	0 versus 0 versus 0	NS	ND

CAG: coronary angiography; CI-AKI: contrast-induced acute kidney injury; Cr: creatinine; CysC: cystatin C; CT: computed tomography; d: day; h: hour; IV: intravenous; kg: kilogram body weight; mg: milligram; mL: milliliter; NAC: N-acetylcysteine; ND: no data; N/2: 0.45% NaCl; OD: once daily; po: per oral route; q12h: every 12 hours; RRT: renal replacement therapy.

**Table 7:** Prospective, randomized clinical trials comparing efficacy of dopamine with placebo and other agents for contrast-induced AKI after angiography prophylaxis.

Authors	Year	Type of procedure and contrast media	Number of patients Intervention versus control	Study protocol		Intravascular volume expansion and NAC protocol	CI-AKI definition	Mean GFR Intervention versus control (mL/min/1.73 m^2^)	Incidence of CI-AKI		RRT requirement Intervention versus control (%)
				Intervention	Control				Intervention versus control (%)	*P* value	
Hans et al. [100]	1998	PAG Iohexol	28 versus 27	Dopamine 2.5 mcg/kg/h 1 hour before and 11 hours after	NSS	None	↑Cr ≥0.5/2 days	42.18 versus 48.8	7.1 versus 28.6	0.026	ND

Kapoor et al. [99]	1996	CAG Urograffin	20 versus 20	Dopamine 5 mcg/kg/h 30 minutes before and 6–8 hours after	None	None	↑Cr ≥25%/1 day ↑Cr ≥25%/3 days	*Cr 1.50 versus 1.52	0 versus 50	ND	0 versus 0

Abizaid et al. [101]	1999	CAG Hexabrix	20 versus 20 versus 20	Dopamine 2.5 mcg/kg/h	(1) None (2) Aminophylline IV 4 mg/kg then 0.4 mg/kg/h	N/2 1 mL/kg/h 12 hours before and after	↑Cr ≥25%/2 days	*Cr 1.9 versus 2.3 versus 1.9	50 versus 30 versus 35	0.60	0 versus 0 versus 5

Stevens et al. [102]	1999	CAG ND	22 versus 21 versus 55	Dopamine 3 mcg/kg/h, Furosemide 1 mg/kg IV before, and Mannitol 12.5 g in D5W 250 mL IV in 2 hours	(1) Dopamine 3 mcg/kg/h and Furosemide 1 mg/kg IV before (2) None	N/2 150 mL/h 6 hours after then adjust to match urine output	↑Cr ≥25%/0.5–2 days	33.73 versus 31.44 versus 30.48	31.8 versus 33.3 versus 30.9	0.98	4.5 versus 4.8 versus 9.1

*Mean GFR data is not available. Data is shown as mean serum creatinine in mg/dL.

CAG: coronary angiography; CI-AKI: contrast-induced acute kidney injury; Cr: creatinine; g: gram; D5W: 5% dextrose solution; h: hour; IV: intravenous; kg: kilogram body weight; mcg: microgram; mg: milligram; mL: milliliter, NAC: N-acetylcysteine; ND: no data; N/2: 0.45% NaCl; PAG: peripheral angiography; po: per oral route; q12h: every 12 hours; RRT: renal replacement therapy.

**Table 8:** Clinical trials comparing fenoldopam with placebo and other agents for prophylaxis of contrast-induced AKI after angiography.

Authors	Year	Type of procedure and contrast media	Number of patients Intervention versus control	Study protocol		Intravascular volume expansion and NAC protocol	CI-AKI definition	Mean GFR Intervention versus control (mL/min/1.73 m^2^)	Incidence of CI-AKI		RRT requirement Intervention versus control (%)
				Intervention	Control				Intervention versus control (%)	*P* value	
Allaqab and et al. [103]	2002	CAG LONICM	38 versus 40 versus 45	Fenoldopam 0.1 mcg/kg/h IV 4 hours before and after	(1) None (2) NAC 600 mg po bid	N/2 1 mL/kg/h 12 hours before and after	↑Cr ≥0.5/2 days	35.5 versus 34.1 versus 36.9	15.7 versus 15.3 versus 17.7	0.919	**Total 1.62

Stone et al. [104]	2003	CAG ND	157 versus 158	Fenoldopam 0.05–0.1 mcg/kg/h IV 1 hour before and 12 hours after	Placebo	N/2 1.5 mL/kg/h 2–12 hours before (1 mL/kg/h if CHF)	↑Cr ≥25%/1–4 days	29.0 versus 29.1	33.6 versus 30.1	0.61	2.6 versus 1.9

Ng et al. [105]	2006	CAG LONICM and IONICM	47 versus 48	Fenoldopam 0.1 mcg/kg/h IV 1-2 hours before and 6 hours after	NAC 600 mg po bid	NSS or D5W 1 mL/kg/h 1-2 hours before and 6–12 hours after	↑Cr ≥25%/1–3 days or ↑Cr ≥0.5/1–3 days	*Cr 1.53 versus 1.46	20.0 versus 11.4	0.40	ND

*Mean GFR data is not available. Data is shown as mean serum creatinine in mg/dL.

**Percentage of RRT requirement data in each group is not available. Data is shown as percentage of RRT requirement in all patients.

bid: twice daily; CAG: coronary angiography; CHF: congestive heart failure; CI-AKI: contrast-induced acute kidney injury; Cr: creatinine; g: gram; D5W: 5% dextrose solution; h: hour; IONICM: isoosmolarity nonionic contrast media; IV: intravenous; kg: kilogram body weight; LONICM: low osmolarity nonionic contrast media; mcg: microgram; mg: milligram; mL: milliliter; NAC: N-acetylcysteine; ND: no data; NSS: normal saline solution; N/2: 0.45% NaCl; po: per oral route; q12h: every 12 hours; RRT: renal replacement therapy.

**Table 9:** Clinical trials comparing theophylline with placebo and other agents for prophylaxis of contrast-induced AKI after angiography.

Authors	Year	Type of procedure and contrast media	Number of patients Intervention versus control	Study protocol		Intravascular volume expansion and NAC protocol	CI-AKI definition	Mean GFR Intervention versus control (mL/min/1.73 m^2^)	Incidence of CI-AKI		RRT requirement Intervention versus control (%)
				Intervention	Control				Intervention versus control (%)	*P* value	
Huber et al. [107]	2002	CAG/PAG Iomeprol	50 versus 50	Theophylline 200 mg IV 30 minutes before	Placebo	ND	↑Cr ≥0.5/2 days	*Cr 2.07 versus 1.92	4 versus 16	0.042	ND

Huber et al. [108]	2003	CAG Imeron	50 versus 50	Theophylline 200 mg IV 30 minutes before	Placebo	ND	↑Cr ≥0.5/2 days	*Cr 1.65 versus 1.72	4 versus 20	0.0138	ND

Dussol et al. [33]	2006	Various procedures Ioxaglate, iobitriodol and iopromide	80 versus 76 versus 77 versus 79	Theophylline 5 mg/kg 1 hour before	(1) NaCl 0.1 g/kg po 2 days (2) NSS 15 ml/kg IV 6 hours (3) (2) NSS 15 mL/kg IV 6 hours and Furosemide 3 mg/kg IV	ND	↑Cr ≥0.5/2 days	33 versus 38 versus 33 versus 34	7.5 versus 6.6 versus 5.2 versus 15.2	ND	0 versus 0 versus 0 versus 0

Huber et al. [109]	2006	Various procedures Imeron	51 versus 50 versus 49	Theophylline 200 mg IV 30 minutes before	(1) NAC 600 mg IV bid (2) Combination	According to underlying disease	↑Cr ≥0.5/2 days	*Cr 1.25 versus 1.25 versus 1.28	2 versus 12 versus 4	0.047 0.53**	2.7

Baskurt et al. [110]	2009	CAG Ioversol	72 versus 72 versus 73	Theophylline200 mg + NAC 600 mg po bid	(1) None (2) NAC 600 mg po bid	NSS 1 mL/kg/h 12 hours before and after	↑Cr ≥0.5/2 days	*Cr 1.47 versus 1.3 versus 1.39	0 versus 6.9 versus 9.6	0.033	0 versus 0

Kinbara et al. [111]	2010	CAG Iopamidol	15 versus 15	Theophylline 250 mg IV 30 minutes before	(1) None (2) NAC 704 mg po bid	NSS 1 mL/kg/h 30 minutes before and 10 hours after	↑Cr ≥0.5/2 days	63.4 versus 63.7 versus 62.4	0 versus 26.7 versus 0	0.0109	0 versus 0

Bilasy et al. [112]	2012	CAG Iopamidol	30 versus 30	Theophylline 200 mg in NSS 100 mL IV 30 minutes before and NAC 600 mg po bid	Placebo and NAC 600 mg po bid	NSS 1 mL/kg/h 12 hours before and after (In case of CHF NSS 0.5 mL/kg/h)	↑Cr ≥25%/3 days or ↑Cr ≥0.5/3 days	58.6 versus 61.8	0 versus 20	0.01	ND

Abizaid et al. [101]	1999	CAG Hexabrix	20 versus 20 versus 20	Aminophylline IV 4 mg/kg then 0.4 mg/kg/h 2 hours before	(1) None (2) Dopamine 2.5 mcg/kg/h 2 hours before	N/2 1 mL/kg/h 12 hours before and after	↑Cr≥ 25%/2 days	*Cr 1.9 versus 2.3 versus 1.9	35 versus 30 versus 50	0.60	5 versus 0 versus 0

*Mean GFR data is not available. Data is shown as mean serum creatinine in mg/dL.

**P value of intervention group compare to combination group.

bid: twice daily; CAG: coronary angiography; CHF: congestive heart failure; CI-AKI: contrast-induced acute kidney injury; Cr: creatinine; g: gram; D5W: 5% dextrose solution; h: hour; IV: intravenous; kg: kilogram body weight; mg: milligram; mL: milliliter; NAC: N-acetylcysteine; ND: no data; NSS: normal saline solution; N/2: 0.45% NaCl; po: per oral route; q12h: every 12 hours; RRT: renal replacement therapy.

**Table 10:** Clinical trials comparing nebivolol with placebo and other agents for prophylaxis of contrast-induced AKI after angiography.

Authors	Year	Type of procedure and contrast media	Number of patients Intervention versus control	Study protocol		Intravascular volume expansion and NAC protocol	CI-AKI definition	Mean GFR Intervention versus control (mL/min/1.73 m^2^)	Incidence of CI-AKI		RRT requirement Intervention versus control (%)
				Intervention	Control				Intervention versus control (%)	*P* value	
Avci et al. [119]	2011	CAG Ioxaglate	55 versus 35	Nebivolol 5 mg po OD 1 week before to 2 days after	Metoprolol 50 mg week before to 2 days after	NSS 1 mL/kg/h 12 hours before and 24 hours after	↑Cr ≥25%/2 days	44.75 versus 43.27	24 versus 33	0.039	ND

Günebakmaz et al. [120]	2012	CAG Iopramide	40 versus 40 versus 40	Nebivolol 5 mg po OD 1 day before and after	(1) None (2) NAC 600 mg po bid	NSS 1 mL/kg/h 6 hours before and 12 hours after	↑Cr ≥25%/2 or 5 days or ↑Cr ≥0.5/2 or 5 days	51.6 versus 47.6 versus 49.8	20 versus 27.5 versus 22.5	0.72	ND

*Number of patient data in each group is not available. Data is shown as total patients in both groups.

**Incidence of CI-AKI for intervention group data is not available. Data is shown as percentage incidence of CI-AKI decrease compared to control group.

CAG: coronary angiography; CHF: congestive heart failure; CI-AKI: contrast-induced acute kidney injury; Cr: creatinine; g: gram; D5W: 5% dextrose solution; h: hour; IV: intravenous; kg: kilogram body weight; mcg: microgram; mg: milligram; mL: milliliter; NAC: N-acetylcysteine; ND: no data; NSS: normal saline solution; N/2: 0.45% NaCl; po: per oral route; q12h: every 12 hours; RRT: renal replacement therapy; tid: thrice daily.
